# Broad-scale recombination pattern in the primitive bird *Rhea americana* (Ratites, Palaeognathae)

**DOI:** 10.1371/journal.pone.0187549

**Published:** 2017-11-02

**Authors:** Lucía del Priore, María Inés Pigozzi

**Affiliations:** INBIOMED Instituto de Investigaciones Biomédicas UBA-CONICET, Facultad de Medicina, Universidad de Buenos Aires, Buenos Aires, Argentina; University of California San Francisco, UNITED STATES

## Abstract

Birds have genomic and chromosomal features that make them an attractive group to analyze the evolution of recombination rate and the distribution of crossing over. Yet, analyses are biased towards certain species, especially domestic poultry and passerines. Here we analyze for the first time the recombination rate and crossover distribution in the primitive ratite bird, Rhea americana (Rheiformes, Palaeognathae). Using a cytogenetic approach for in situ mapping of crossovers we found that the total genetic map is 3050 cM with a global recombination rate of 2.1 cM/Mb for female rheas. In the five largest macrobivalents there were 3 or more crossovers in most bivalents. Recombination rates for macrobivalents ranges between 1.8–2.1 cM/Mb and the physical length of their synaptonemal complexes is highly predictive of their genetic lengths. The crossover rate at the pseudoautosomal region is 2.1 cM/Mb, similar to those of autosomal pairs 5 and 6 and only slightly higher compared to other macroautosomes. It is suggested that the presence of multiple crossovers on the largest macrobivalents is a feature common to many avian groups, irrespective of their position throughout phylogeny. These data provide new insights to analyze the heterogeneous recombination landscape of birds.

## Introduction

Crossover (CO) is fundamental for sexual reproduction and results in the reciprocal exchange of large chromosome segments between homologues that increases genetic variability in populations. Among vertebrates, the number and distribution of crossovers were studied largely in mammals, either with cytological visualization of crossover markers or linkage analysis [1, 2]. It was shown that the number of COs is correlated with the number of chromosome arms or the number of chromosomes [3, 4] and mean recombination rates span an order of magnitude, from 0.2 cM (centimorgans)/Mb to 1.6 cM/Mb [5]. Recombination analyses in closely related species also indicated the existence of a strong phylogenetic signal in average recombination rates [6] and suggested that deeper lineages in the mammalian tree of life such as Marsupialia and Afrotheria have lower recombination rates than do species that have diverged more recently [7].

In birds, cytogenetic studies revealed the presence of multiple CO events along the largest macrobivalents in the chicken, the preferential location of recombination events toward the ends of chromosomes in zebra finches and the limited variation in recombination rates between sexes in pigeons and quails [8–10]. Genetic linkage analysis in chickens showed that recombination rates were unusually high in this species, especially when compared to recombination rates in mammals [11–13]. Fine-scale recombination maps in the zebra finch and in natural population of other passerine birds suggest that COs are strongly localized towards the ends of chromosome with rare CO events at the center chromosome regions [14–18]. Altogether, cytological and linkage studies, show that recombination events distribute heterogeneously along chromosomes varying from few highly localized CO events to multiple exchanges with alternating peaks and valleys of recombination along chromosomal arms [9, 19–22].

Most research of recombination in birds have focused on domestic Galloanserae and a number of passerine birds [22, 23], with limited information from other avian orders [20]. The karyotype of birds has remained more or less intact during 100 million years of avian evolution, with the ancestral karyotype being very similar to that of the chicken [24, 25], favoring a comparative approach of CO distribution and its relationship with chromosome morphology and DNA content. Therefore, exploring other, more distantly related species is essential to compare the recombination rates and CO distribution across avian phylogeny. For these reasons, the present analysis is focused on the CO pattern in the Greater rhea (*Rhea americana*), a ratite bird native to South America that belongs to the primitive Superorder Palaeognathae. We use the immunolocalization of the protein MLH1 for *in situ* imaging of COs along bivalents in pachytene oocytes. CO detection using antibodies against MLH1 protein has been performed predominantly in mammals, but the method has also been used in several species of birds [23]. This method allows scoring the number of COs and their distribution on chromosome arms in whole meiotic cells because the MLH1 protein forms discrete foci detectable by immunofluorescence along the linear synaptonemal complexes (SCs), which represent synapsed bivalents at pachytene [26–30].

In this paper, for the first time in the Greater rhea we analyze the total number of CO in the complete set, the distribution of COs on the largest identifiable bivalents and estimate the recombination rates for the whole genome and for individual bivalents. Our observations suggest that the presence of multiple COs along macrobivalents and the global recombination rate observed in the Greater rhea are representative of the ancestral CO landscape of birds.

## Materials and methods

### Biological material

Eggs from *R*. *americana* were obtained from the farm Pampa Cuyen located in Buenos Aires Province, Argentina. At the time of the study, rheas had been bred in captivity for 10 years with the introduction of animals from the wild in three instances [31]. The meiotic cells used in the analysis were obtained from three female embryos. Handling and euthanasia of birds were performed as per protocols approved by the Animal Care and Use Committee of the University of Buenos Aires School Of Medicine (EXP-UBA 005192/13, Res 2350/13) following all institutional and national guidelines for the care and use of farm and laboratory animals.

### Synaptonemal complex spreads, immunostaining and image acquisition

In birds, oocytes enter into meiosis before hatching and reach the pachytene stage at different developmental stages. In the Greater rhea, previous analyses showed that pachytene oocytes are found in embryos one week to ten days before hatching [32]. To make SC spreads, small portions of the only functional (left) ovary were minced in 100 mM sucrose to obtain a clump-free cell suspension. About 30 μl of this suspension were dropped on glass slides covered with a thin film of 1% paraformaldehyde containing 0.1% Triton X-100. Slides were left in a humid chamber at room temperature for 2 hours, then washed in a 0.08% Photoflo (Kodak) solution and air dried. Unstained slides were examined under phase microscopy, and only slides on which the cells were well spread were immunostained. Immunostaining was performed as previously described [33] using mouse anti-MLH1 (at 1: 30, BD Pharmingen), CREST human antiserum (at 1:100, Roquel Laboratories, Buenos Aires, Argentina) that binds to kinetochores and rabbit anti-SMC3 (Chemicon, Millipore, at 1:1000) that labels the SCs. The secondary antibodies were TRITC-labeled goat anti-rabbit, Cy3-labeled donkey anti-human and FITC-labeled goat anti-mouse (Jackson ImmunoResearch). Immunostained spreads were scanned with 100X magnification objective at a fluorescence microscope equipped with appropriate filter sets for each fluorochrome. Individual images for red and green fluorescence were acquired using an Olympus DP73 CCD camera, corrected for brightness and contrast and merged using Adobe Photoshop CS5. C-banding [34] or immunostaining with an antibody against-H3K9me3 (Abcam) were employed to observe heterocromatin on mitotic and meiotic chromosomes, respectively.

### Analysis of MLH1 distribution on SCs

For each SC, the position of each MLH1 focus was recorded as a relative distance from the centromere, using MicroMeasure (http://rydberg.biology.colostate.edu/MicroMeasure). Measurements of the SC set were scored in a sample of 94 pachytene nuclei immunostained for SCs and centromeric proteins. The procedure to build the MLH1-focus distributions along SC arms is based on the method initially employed to map late recombination nodules in plants and other organisms [10, 35]. To obtain an average absolute length for each identified SC, the average relative length of each SC was determined, and then multiplied by the average absolute length of a complete SC set of rhea (278.7 μm). MLH1-crossover maps were obtained from 85 nuclei that met the same criteria employed in previous analyses [8]. The relative position of each MLH1 focus was multiplied by the average absolute length for the appropriate SC to obtain the absolute (micrometer) position of each focus (S1 File). The data for each one of the six largest autosomal SCs and the ZW pair were pooled and graphed in histogram form to demonstrate the pattern of MLH1 distribution. Because each CO marked by an MLH1 focus results in 50% recombinant progeny [36], the total length of the genetic map is equal to the average number of MLH1 foci per genome multiplied by 50 centimorgans (cM). Similarly, the genetic map length of each identified bivalent is equal to the average number of foci on the corresponding SC multiplied by 50.

### Estimation of genome and chromosome sizes

The haploid DNA content of *R*. *americana* is about 1.46 pg (T.R. Gregory, personal communication). Since the DNA content in pg can be converted to millions of base pairs (Mb) multiplying by 0.978 X 10^9^ [37], the estimated genome size of the Greater rhea would be then 1428 Mb. The size in Mb of the six largest autosomes was predicted from the relative length of their SCs multiplied by the haploid genome content, as previously done in other organisms [38]. This procedure assumes that SC length is proportional to genome size and it is supported by the fact that SC length is a very good predictor of the Mb chromosome size in the chicken [19].

## Results and discussion

### The SC karyotype, microbivalent morphology and identification of the ZW pair

**Fig 1:**
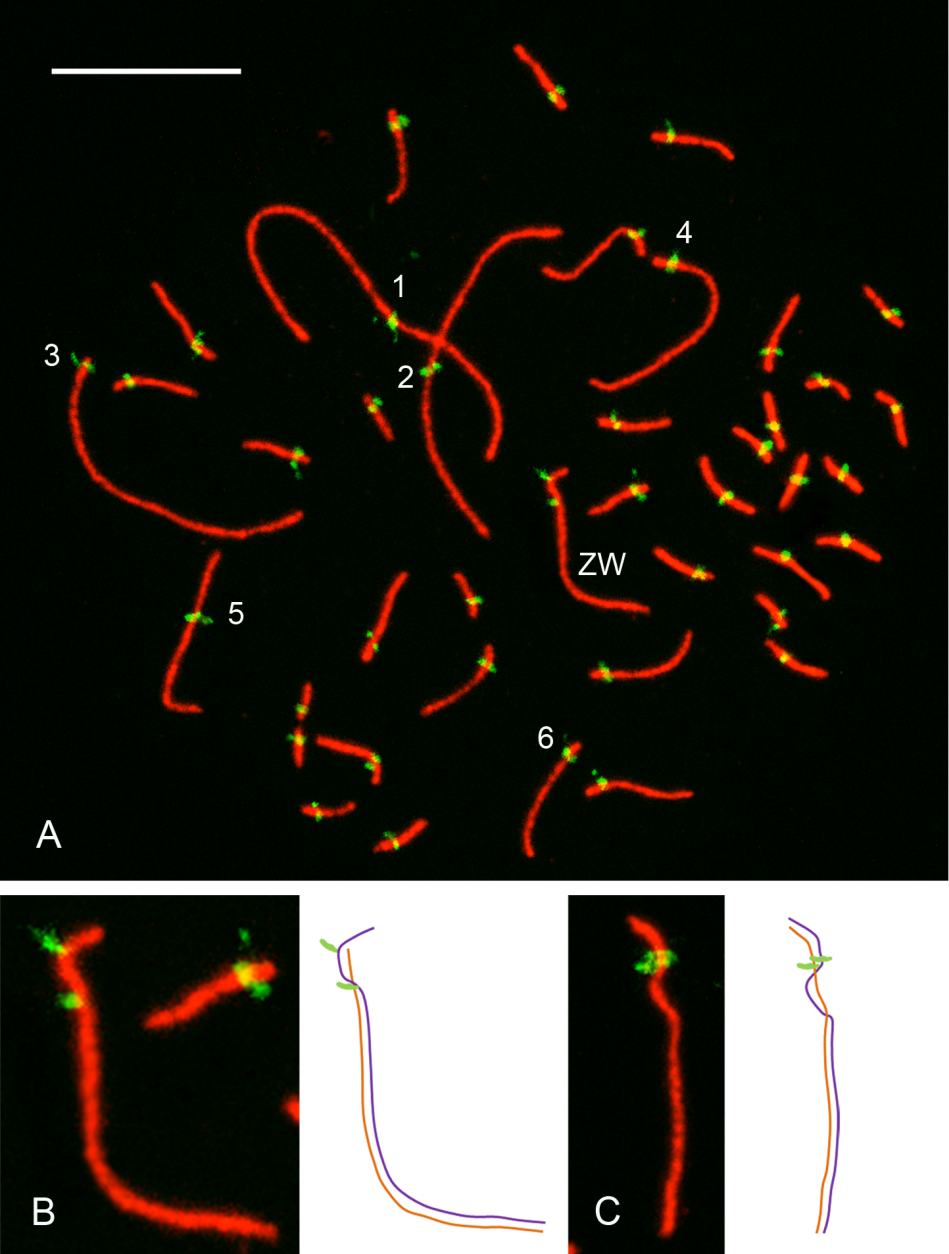
Autosomal SCs and the ZW pair during pachytene. A. SC spread from an oocyte of the Greater rhea at pachytene showing the immunostained synaptonemal complexes (red) and centromeres (green). Bar = 10 μm. The number next to the centromeric signals identifies the largest autosomal SCs. B. ZW pair showing misaligned centromeric signals indicating an early stage of synaptic adjustment. C. ZW pair after completion of length adjustment of the Z and W axes. Drawings of ZW synaptic configurations are shown to the right of images B and C.

The Greater rhea (2n = 80) has a typical avian karyotype with chromosome sizes that follow a continuous distribution. The six largest autosomal pairs, discernible on the basis of their size and centromere position, are called here macrochromosomes. Their respective SCs are also distinct in pachytene spreads (Fig 1) and will be referred to as macrobivalents, while the rest of the autosomal bivalents (7–39) are considered microbivalents in order to simplify the descriptions throughout the text. Measurements show a very good morphological agreement between the mitotic macrochromosomes and their respective SCs: macrobivalents 1, 2 and 5 are submetacentric while macrobivalents 3, 4 and 6 are acrocentric (S1 Fig; S1 Table; S1 File).

Most microbivalents have subterminal centromeric signals, but at least ten of them have more distinctive short arms and can be classified as metacentric or submetacentric (Fig 1A; S1 Fig). Previous SC analyses showed that centromeres on microbivalents have predominantly a near-terminal position in most birds [10, 39–41], with the notable exception of numerous metacentric microbivalents in the common quail [9]. It has been proposed that the dissimilar centromeric position in quails compared to the chicken is due to accumulation of repetitive sequences on the short arms of quail microchromosomes [42]. In C-banded mitotic metaphases from rhea 10 pairs of microchromosomes appear almost completely formed by heterochromatin (S2 Fig). This number of heterochromatic microchromosomes is similar to the number of bi-armed microbivalents supporting the idea that heterochromatin acquisition/loss might be related to this centromere repositioning. The presence of bi-armed microbivalents in distantly related species of birds, such as quails and the Greater rhea indicates that non-acrocentric microbivalents arose independently in different lineages and more cases could be uncovered by examination of SCs in pachytene oocytes and spermatocytes.

Like other ratite birds, the Greater rhea has a slightly heteromorphic ZW pair with a large recombining or pseudoautosomal region (PAR) [32]. Following synapsis, the heteromorphic Z and W meiotic axes of birds become almost equal in length due to the progressive adjustment of the individual axes [43]. This process of axial adjustment is also present in the nearly homomorphic sex chromosomes of rheas [32]. In the present analysis the centromeric signals on the Z and W axes were found either misaligned or in register, representing sequential stages before and after the axial adjustment, respectively (Fig 1B and 1C). Even after the adjustment, the ZW bivalent could be singled out based on relative size, centromere position and the distinctive “wavy” end corresponding to the non-recombining segment of the bivalent (Fig 1).

### Genetic map length and crossover maps for individual bivalents inferred from MLH1-focus counts

**Fig 2:**
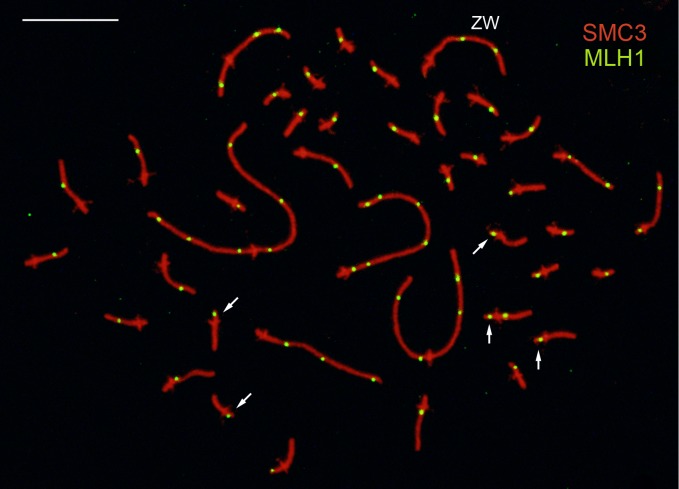
MLH1 foci in the Greater rhea. The MLH1 foci appear as distinct green dots along the SCs. Centromeres (detected in red) protrude from the linear SCs. Arrows point to some of the microbivalents with a single MLH1 focus on the short arm. The ZW bivalent has two foci on the long arm. The differential, non-recombining region of the bivalent adopts a wavy contour after the axial adjustment. Bar: 10 μm.

**Fig 3:**
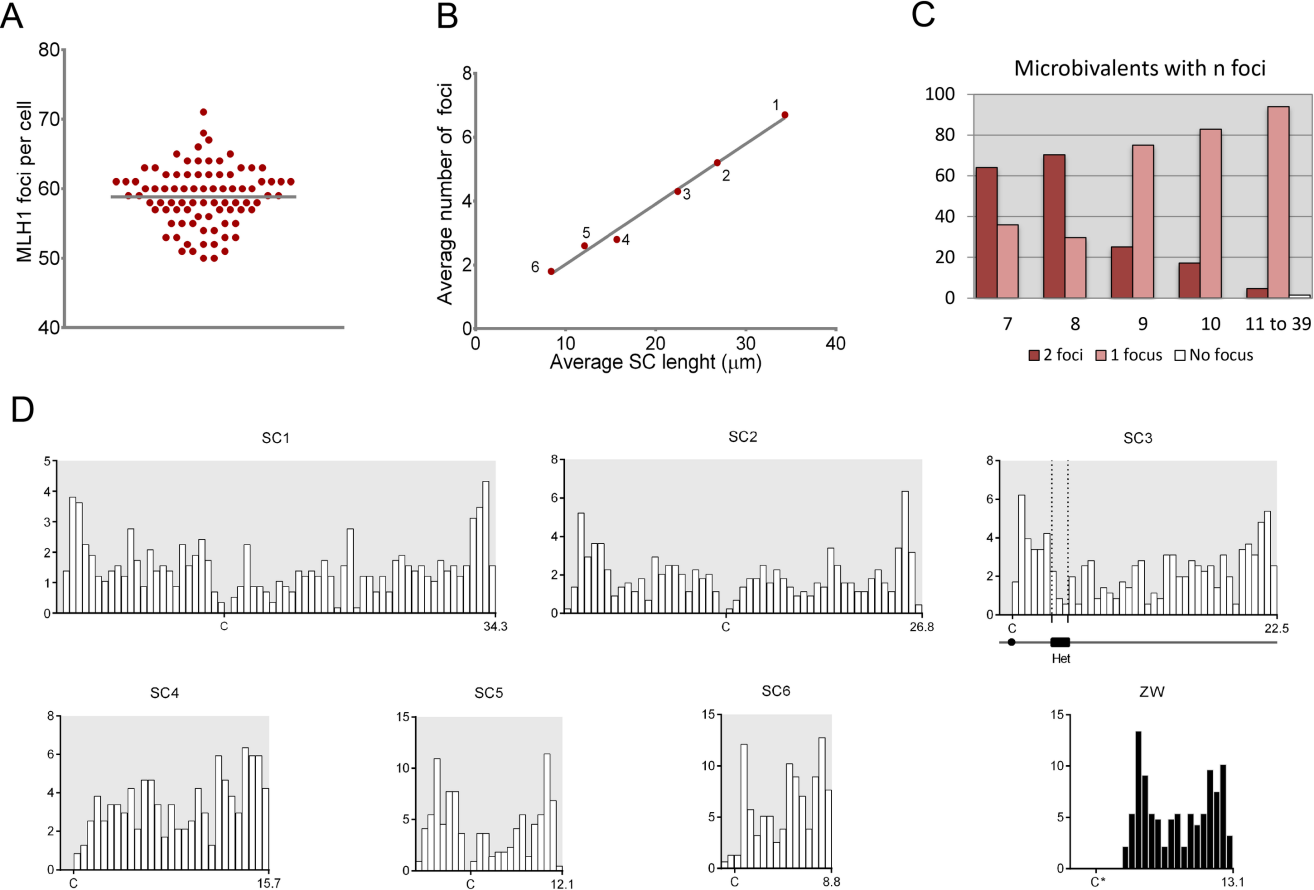
Analysis of crossing over in rhea oocytes. A. In the graph each dot represents a single pachytene nucleus with n foci. The horizontal line marks the average number of foci found in 85 oocytes. B. Relationship between average SC length (μm) and the average number of MLH1 foci on the six largest autosomal bivalents. C. MLH1 foci on microbivalents. The numbers below the X-axis indicate the rank length of the SC. With the exception of microbivalents ranking 7th and 8th, most micro-SCs show a single focus. D. Distribution of MLH1 foci on individual SCs. For each SC, the X-axis indicates the relative positions of the MLH1 foci on the short (left) and long (right) arms. Each histogram represents the distribution of MLH1 foci along the six largest autosomal SCs and the ZW pair. The bin width in each histogram represents a fraction of the total length of each SC, and it is equivalent to 0.5 μm. “C” = centromere. Het: heterochromatin.

The total number of MLH1 foci per nucleus was scored in a sample of 85 nuclei with complete SC sets (Fig 2; S1 File). The total number of foci in the autosomal set ranged between 50 and 71 (Fig 3A), with an average of 58.8, totaling 2940 cM for the autosomal set (Table 1). For the complete female genome, 110 cM should be added since the ZW pair has an average of 2.2 foci (110 cM), giving a total map length of 3050 cM. Altogether 3400 bivalents, including the ZW pair, were analyzed for MLH1 foci. Of these, less than 1% lacked an MLH1 focus and in all cases they were microbivalents ranking 11 to 39. At present no alternative CO markers have been analyzed on autosomal SCs of the Greater rhea, so it is not possible to determine if these bivalents represent true cases of recombination failure or if the absence of MLH1 was due to methodological reasons. This frequency of bivalents without a focus is within the limits of variation found in MLH1 studies in other birds and also in mammals [19, 44]. MLH1-focus numbers are comparable to chiasmata at least in pigeons, quails and chickens, favoring the idea that MLH1 protein signals most COs in birds [45]. For these reasons, we consider that gross underestimation of CO frequencies due to the present methodology can be ruled out.

**Table 1:** Number of MLH1 foci and crossover rates for individual and grouped SCs.

SC	MLH1 foci				Genetic length(cM)^a^	Physical size(Mb)^b^	CO rate(cM/Mb)
	Mean	SD	Min-Max	≥ 3 COs (%)^c^			
**1**	6.8	1.2	4–9	100	340	175.6	1.9
**2**	5.2	0.9	3–8	99.9	260	134.2	1.9
**3**	4.3	1.0	2–6	99.9	215	112.8	1.9
**4**	2.8	0.7	2–5	64.3	140	80.0	1.8
**5**	2.6	0.6	2–4	53.0	130	61.4	2.1
**6**	1.8	0.6	1–3	4.4	90	43.4	2.1
**7–39**	35.3	2.5	1–2	0	1765	748.8	2.4
**ZW**	2.2	0.5	1–3	22.6	110	65.7	1.7/2.1^d^
**Total**	61	4.4			3050	1428	2.1

^a^ Mean number of MLH1 foci x 50; (1 CO = 50 cM)

^b^ Estimated from the haploid DNA content and SC relative lengths

^c^ Percent bivalents with 3 or more MLH1 foci

^d^ CO rate at the pseudoautosomal region (80% of bivalent size)

As expected on the basis of previous analysis of MLH1 foci in birds, longer SCs average more MLH1 foci than do shorter SCs (Table 1). A strong positive correlation is observed when the mean SC length for individual bivalents is plotted against the mean number of MLH1 foci (Fig 3B; *y* = 0.20*x* + 0.13; *r*^*2*^ = 0.99). This relationship indicates that SC length measures genetic distance and it is similar to observations in oocytes and spermatocytes of different birds and mammals [8, 20, 30, 41, 46]. Among microbivalents, the presence of two foci was frequent in bivalents ranking 7 and 8, while the shortest SCs had predominantly one focus (Fig 3C). Further analysis of MLH1 foci on microbivalents is presented in a separate section (*MLH1 foci on microbivalents*).

The distribution of MLH1 foci for individual macrobivalents shows that CO events lack a strong localization (Fig 3D). MLH1 foci were found in most SC intervals (equivalent to 0.5 μm) with the exception of the short arms of the acrocentric bivalents 3 and 4, some pericentromeric intervals and the differential region of the ZW pair. Even though, distal regions show higher recombination levels compared to interstitial ones, the distribution of COs is not confined to chromosome ends. A region with low frequency of foci on SC 3 was linked to the presence of heterochromatin at the same relative position, as evidenced by C-banding on mitotic chromosomes (S2 Fig). This interstitial heterochromatin affects negatively the occurrence of COs compared to the flanking euchromatic regions but it does not suppress CO completely. In the same number of meioses, the heterochromatic SC arms of biarmed microbivalents do not show MLH1 foci (see next section), pointing to the complex interplay between heterochromatin and crossing over [47].

There is a regular presence of three or more MLH1 foci on the largest macrobivalents in the Greater rhea (Table 1). Similar observations were reported in four species of Galloanserae and also in pigeons, with species-specific distributions of foci along chromosomal arms [45, 48]. This occurrence of multiple CO events per macrobivalent, however, is not shared by all birds. MLH1-focus mapping in zebra finches showed that COs are mainly localized towards chromosome ends, with large recombination desserts at the center of chromosomes in males and females [9]. This broad-scale recombination pattern in zebra finches reflects the distribution of hotspots, as shown by fine-scale recombination analysis in the same species [49]. Linkage disequilibrium studies and linkage mapping established that the localized CO pattern first observed in zebra finches also exists in other passerine birds such as flycatchers and the great tit [14, 50]. So far, there is no molecular basis that explains these highly heterogeneous recombination patterns along avian macrochromosomes. In vertebrates such as rodents and primates, CO distribution and frequencies are partially dependent on the function of the zinc finger protein PR domain-containing 9 (PRDM9) that establish an epigenetic signature at recombination hotspots [51]. PRDM9 is absent in birds and recombination hotspots are associated with functional sequences [52]. Because genomic and karyotype features are largely conserved in birds and hotspots are considered stable in birds compared to mammals, it was speculated that small differences in recombination landscapes between closely related species could be explained by epigenetic changes at hotspots during avian evolution [53]. CO number and distribution, however, do not correlate solely on chromatin marks but also involve mechanistic factors. The structure of the chromosome axis and the chromosome dynamics during meiosis also affect proper CO distribution, as evidenced by the requirement of proteins involved in chromosome movements and the interaction of telomeres with the nuclear envelope [54–57]. In birds, the existence of species with multiple CO events and species with few localized COs on macrobivalents, offers an opportunity to look for the regulatory basis of CO distribution in higher vertebrates.

### Heterochromatin content and recombination rates in microbivalents

**Fig 4:**
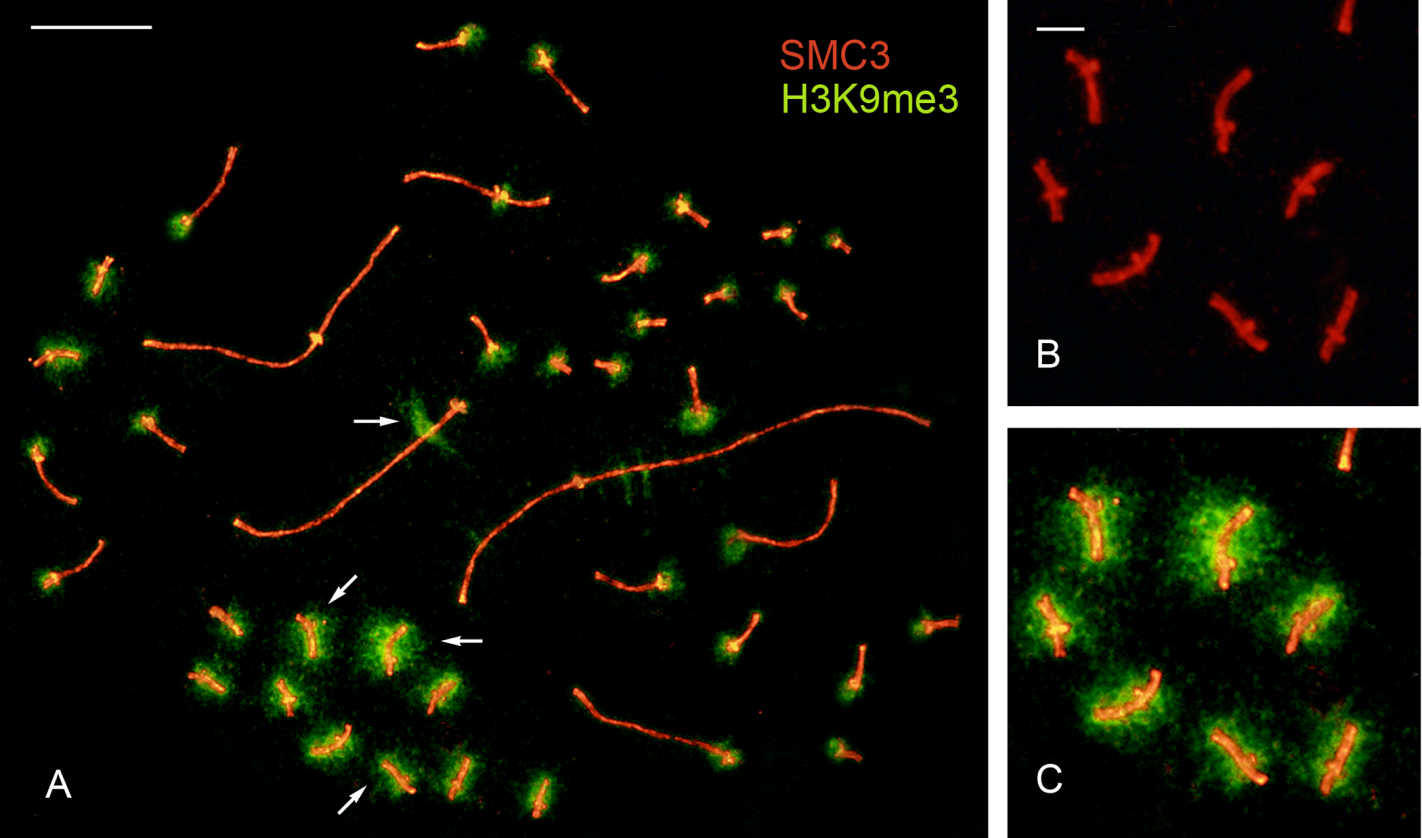
Heterochromatic microbivalents in the Greater rhea. A. Pachytene nucleus immunostained for SMC, centromeres and the heterochromatin marker H3K9me3. Arrows point to some of the heterochromatic microbivalents and to the interstitial heterochromatin in SC 3. Bar: 10 μm. B-C. Notice that heterochromatin marks on the bi-armed microbivalents do not extend beyond the tip of the short arms. Bar: 1 μm.

As explained above, the number of bi-armed microbivalents in SC spreads is similar to the number heterochromatic microchromosome pairs observed in mitotic metaphases (S1 Fig; S2 Fig). These heavily heterochromatic microchromosomes are revealed in SC spreads immunostained with anti-H3K9me3 (Fig 4). In bi-armed microbivalents, the H3K9me3 labeling does not extend towards the telomere of the short arm; instead this labeling covers the short arm of acrocentric microbivalents (Fig 4). MLH1 analysis shows that the submetacentric microbivalents had a single focus on the short arm in almost every cell (Fig 2). Only exceptionally, two foci appear on one of these microbivalents: one on the short arm and the other on the long arm, very close to the centromere. We infer that crossing over is constrained to the short arm because of the presence of heterochromatin on the long arm. Consequently, a substantial amount of DNA is not subject to genetic recombination in these microbivalents resulting in considerably higher recombination rates than those estimated for all microbivalents (Table 1). Most avian microchromosomes have a single CO event and, depending on the species, a number of them are enriched in repetitive sequences and constitutive heterochromatin [58–60]. In microchromosomes that are mostly euchromatic, the single CO might take place at variable points of the DNA sequence. Instead, in largely heterochromatic microchromosomes the CO will be restricted to a small euchromatic segment. The presence of a variable number of heterochromatic microchromosomes with a single, localized CO event can explain the large variations of recombination rates (3–14 cM/Mb) observed in the smallest chromosomes of the chicken and other birds [13, 22].

### Pseudoautosomal vs. autosomal recombination rates

The Z and W chromosomes of ratites are largely undifferentiated despite avian sex chromosome evolution was initiated >100 Mya [61]. In the Greater rhea, the PAR comprises the distal 80% of the long arm of the W chromosome (Fig 3), that is, the segment where MLH1 foci occur along the ZW bivalent. The average number of MLH1 foci along this segment is 2.2 (Table 1), and does not differ significantly to the number of recombination nodules found in a previous analysis in this species [32] (t = 0.4198, df = 146; P = 0.6753). The size of the PAR estimated from the stretch of the SC bearing foci is 52.5 Mb. This size compares fairly well with those estimated in other ratites, such as ostriches and emus where the PAR sizes are 63.6 and 65.5 Mb, respectively [62]. The ZW pair of the Greater rhea shows higher levels of differentiation among ratites [63], so a smaller size of the PAR can be expected. We found that the average recombination rate across the PAR is 2.1 cM/Mb, similar to those of autosomal pairs 5 and 6 and only slightly higher compared to other macroautosomes (Table 1). Data about crossing over in the ZW pair of ratites are scarce. Janes et al [64] compared the recombination rates between 14 pseudoautosomal and 8 autosomal loci derived from emu bacterial artificial chromosome (BAC) clones. They concluded that recombination rates are slightly higher and linkage disequilibrium is somewhat lower in the PAR than in autosomal loci. These conclusions however, should be viewed cautiously because of the small size of the sampled data set that comprised 4.82 kb. Clearly, more analyses are needed to determine if crossing over in the large PAR of ratites exhibit differential features compared to autosomes. One approach could be to compare recombination in the PAR with the equivalent region in the ZZ pair of males, but if overall rates of recombination are different between sexes, this might make it difficult to discover properties specific to the PAR.

## Supporting information

S1 Fig Mitotic chromosomes and synaptonemal complexes in *Rhea americana*. A. DAPI-stained mitotic metaphase from a female. B. Comparison of the macrochromosomes with their respective SCs. The first six autosomal SCs and the ZW bivalent of the oocyte in Fig 1 (manuscript) were digitally straightened to show their distinctive lengths and centromere positions. C. Microbivalents of the same oocyte arrayed by size. The bi-armed microbivalents are identified as sm and m, according to the relative length of the short arm.
(TIF)

S2 Fig Heterochromatin distribution on the chromosomes of the Greater rhea. A. Representative C-banded mitotic metaphase. The arrows point to the interstitial heterochromatin on chromosome 3. B. Numbers in red show the total chromosome count (2n = 80). Numbers in blue are the count of heterochromatic microchromosomes. Both totals were obtained using the Count tool in Adobe Photoshop CS5.
(TIF)

S1 File Datasets to produce graphs and tables. (XLSX)

S1 Table Comparisons of the relative lengths and centromere index between synaptonemal complexes and mitotic chromosomes. (DOCX)
