# Supplementary figures and images for: Broad-scale recombination pattern in the primitive bird Rhea americana (Ratites, Palaeognathae)
Source: PLoS One. 2017 Nov 2;12(11):e0187549. doi: 10.1371/journal.pone.0187549 (PMC5667853; doi:10.1371/journal.pone.0187549)

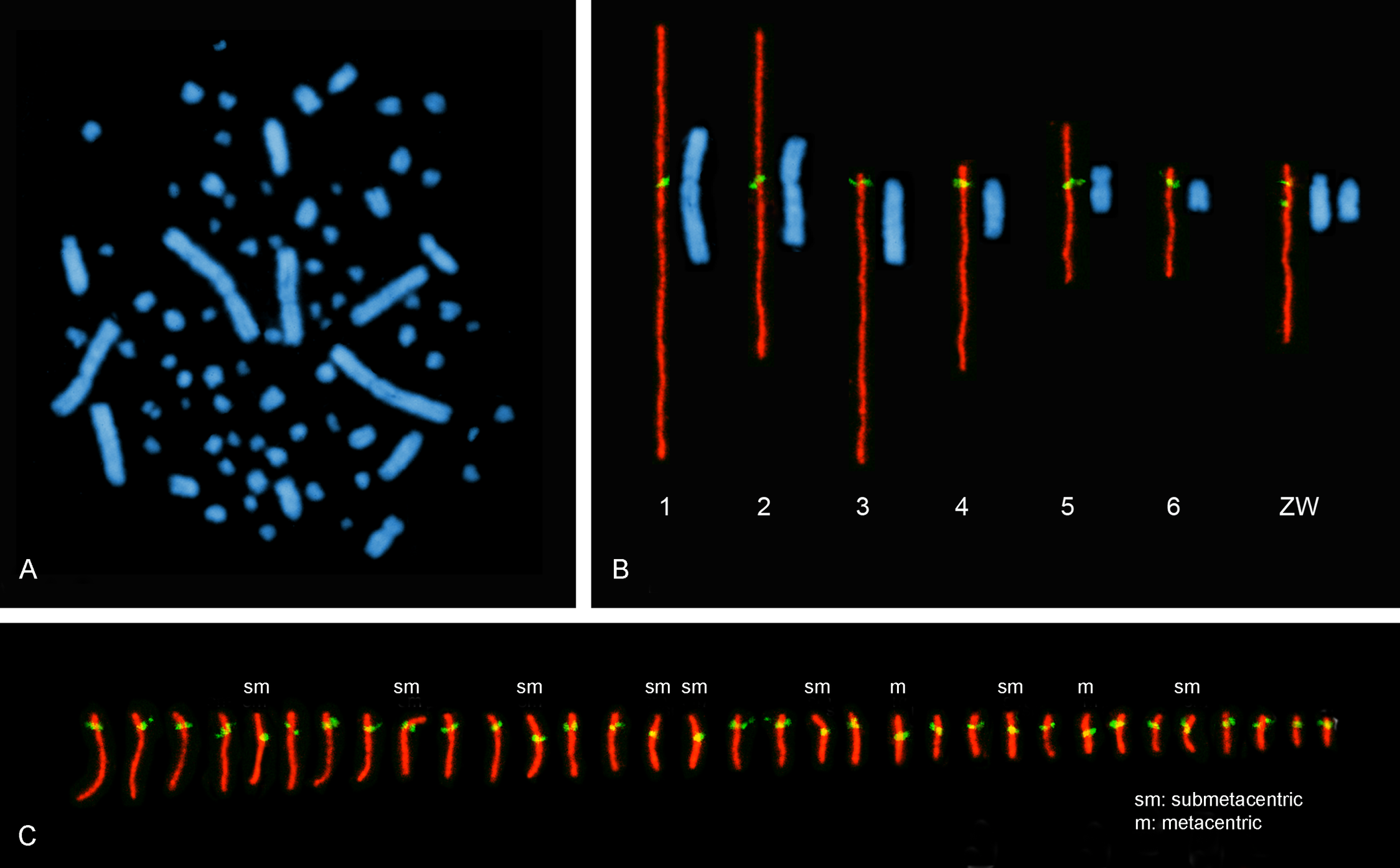

Supplement: S1 Fig — A. DAPI-stained mitotic metaphase from a female. B. Comparison of the macrochromosomes with their respective SCs. The first six autosomal SCs and the ZW bivalent of the oocyte in Fig 1 (manuscript) were digitally straightened to show their distinctive lengths and centromere positions. C. Microbivalents of the same oocyte arrayed by size. The bi-armed microbivalents are identified as sm and m, according to the relative length of the short arm. (TIF) [file pone.0187549.s001.tif]

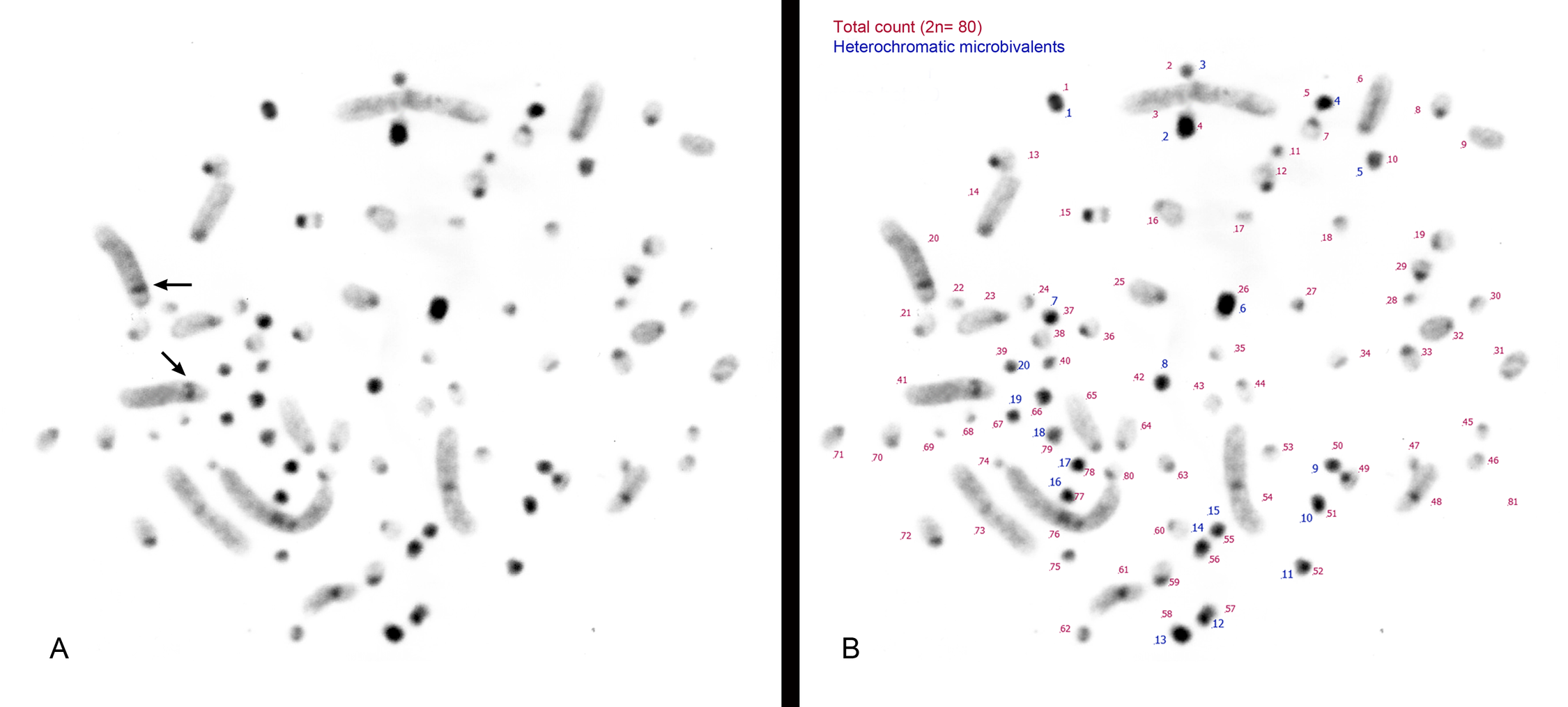

Supplement: S2 Fig — A. Representative C-banded mitotic metaphase. The arrows point to the interstitial heterochromatin on chromosome 3. B. Numbers in red show the total chromosome count (2n = 80). Numbers in blue are the count of heterochromatic microchromosomes. Both totals were obtained using the Count tool in Adobe Photoshop CS5. (TIF) [file pone.0187549.s002.tif]
