# Supplementary material for: Broad-scale recombination pattern in the primitive bird Rhea americana (Ratites, Palaeognathae)
Source: PLoS One. 2017 Nov 2;12(11):e0187549. doi: 10.1371/journal.pone.0187549 (PMC5667853; doi:10.1371/journal.pone.0187549)
Supplement: S1 Table — (DOCX) [file pone.0187549.s004.docx]

| **Chr** | **Synaptonemal complexes^a^** | | | | **Mitotic chromosomes** | | | |
| --- | --- | --- | --- | --- | --- | --- | --- | --- |
|  | **Relative length (%)^c^** | | **Centromere index (%)^d^** | | **Relative length (%)^c^** | | **Centromere index (%)** | |
|  | Mean | SD | Mean | SD | Mean | SD | Mean | SD |
| **1** | 12.3 | 2.3 | 37.4 | 2.1 | 13.7 | 1.0 | 38.0 | 1.7 |
| **2** | 9.4 | 1.7 | 44.0 | 2.6 | 9.9 | 0.6 | 44.6 | 1.8 |
| **3** | 7.9 | 1.1 | 4.0 | 0.6 | 8.5 | 0.5 | 4.4 | 0.7 |
| **4** | 5.6 | 1.1 | 6.3 | 0.2 | 5.5 | 0.4 | - | - |
| **5** | 4.3 | 0.6 | 35.5 | 1.7 | 4.7 | 0.3 | 38.0 | 1.5 |
| **6** | 3.2 | 0.6 | 12.0 | 1.2 | 3.2 | 0.3 | 10.0 | 1.3 |
| **7-39** | 52.7 | 2.5 | - | - | 51.2 | 2.1 | - | - |
| **ZW^e^** | 4.6 | 0.6 | 14.9 | 2.2 | 4.4 | 0.4 | 12 | 1.8 |
